# Will Influenza Return?

**Published:** 1919-07-05

**Authors:** 


### WILL INFLUENZA RETURN?

Although the medical Press has, except for further and more definite publications concerning the suggested filter-passing virus, been lately more or less silent on this question, the pause should by no means suggest to the country that epidemic danger is necessarily over and done with. The first idea that summer has freed us from infection, and that we are safe until the seasons change, is largely negatived when one remembers that in the past two years the epidemics were definitely unrelated to the period of the year. Microbic disease of this type has always been known to run in alternating periods of rising and falling virulence. At present it has fallen to its lowest ebb, but without doubt there will be at least a tendency to return before many months are past.

This of the germs themselves, but the effect upon the community, the incidence and mortality of the disease are quite another matter. It is very hard to give a definite forecast when the degree of severity of any disease as actually seen among the people depends, as in this instance, on so many and various factors. Less mental strain and war worry, more and better food, should go far towards making us less susceptible, and conditions of living are certainly daily becoming more favourable and less liable to give the unique opportunities of mass infection which occurred in war time. Also, we are both forearmed and forewarned by the distressing experiences of the past, and with a now considerable increase in the number of medical men available for private and institutional practice, much should be done towards prophylaxis before or at the first sign of an epidemic.

The methods of hygiene, careful living, all the old advice of going to bed and treating the malady seriously, of course hold good; but, though the employment of artificially produced immunity has been scorned in many quarters, yet one feels there was sufficient evidence collected in 1918 that use of protective vaccines prepared from the mixed strains of those organisms repeatedly found responsible for at least the complications of influenza, went a long way towards shielding communities not already infected. As treatment these injections were disappointing, but for prevention far greater success can be claimed. We had the example of many camps in England, particularly that of the New Zealand contingent, where, during the severest outbreaks, relative immunity prevailed. Several large public schools successfully adopted the same measures, and one does not need to record the details of the well-known vaccination campaigns in America. Absolute immunity is not to be expected. Out of every thousand treated some are immune, some suffer from the disease in a milder form, and others may get little or no apparent protection; but the figures of last year point to an almost complete cessation of the death-rate among protected peoples. In view of the fact that our best general methods of prophylaxis and treatment so signally failed last winter, it is a suggestion, practical and, we think, of probable value, that any signs of a return of influenza should be greeted by the widespread use of, at first small, then increasing doses of mixed vaccine in all instances where exposure to possible mass infection is unavoidable.

